# What Do Adolescents See on Social Media? A Diary Study of Food Marketing Images on Social Media

**DOI:** 10.3389/fpsyg.2019.02637

**Published:** 2019-11-22

**Authors:** Yara Qutteina, Lotte Hallez, Nine Mennes, Charlotte De Backer, Tim Smits

**Affiliations:** ^1^Institute for Media Studies, Faculty of Social Sciences, KU Leuven, Leuven, Belgium; ^2^Department of Communication Sciences, Faculty of Social Sciences, University of Antwerp, Antwerp, Belgium

**Keywords:** social media, influencer, adolescents, diary study, marketing, food, eating

## Abstract

Food marketing influences eating preferences and choices, especially among adolescents, contributing to the rise of overweight, obesity, and other chronic health disorders. Recent social media advancements have provided food marketers with platforms to reach out to many in more personal and authentic ways as compared to classical media advertising. Such personalized and borderless social media platforms allow marketers to easily use owned, paid, and earned (word-of –mouth) marketing strategies, including paid and non-paid influencers to reach younger target audiences. This study therefore aims to explore food messages adolescents (12–18 years old) encounter on social media, and assess these messages for their sources, the presence of core and non-core food, and the marketing strategies employed. To attain an in-depth understanding of the food messages that adolescents are continuously exposed to, we carried out a diary study with 21 Flemish adolescents who took screenshots of food images they encountered on their social media platforms for the duration of one week. A quantitative and qualitative content analysis of 611 images revealed that adolescents are mostly exposed to messages of non-core (67% of images) and branded (49% of images) food, often (49% of images) presented in association with a social context such as hanging around with friends, eating at restaurants and celebrating with food. Adolescents often encounter branded food images through peers and social media influencers, the majority of which are part of earned (49% of branded images) or paid (40% of branded images) media food marketing. This research provides an in-depth understanding of the social media messages that adolescents encounter on a daily basis and sheds light on food norms typically communicated on social media by marketers, peers, and influencers. Study findings highlight prominent social media food messages that should be tested for their persuasiveness, providing insights for future research that aims to assess the effects of social media food marketing on adolescents. Based on the study findings, we call for relevant policy actions that address current social media marketing strategies targeted at adolescents.

## Introduction

Media food marketing, such as food ads and sponsorships seen on television (TV) and magazines, influences eating preferences and choices, especially among minors (Lobstein et al., 2015). A recent review and meta-analysis demonstrates that media food marketing, particularly traditional media (such as TV) food marketing, influences adolescent eating cognitions, attitudes and behaviors (Qutteina et al., 2019). Media food marketing increases the consumption of fried foods, sweets, and other foods that are high in energy and low in nutrients (Qutteina et al., 2019), hereafter referred to as non-core foods (Kellet et al., 2014; Toumpakari et al., 2016). Increased consumption of non-core food is associated with a range of chronic disorders and diseases (such as obesity, cardiovascular disease, cancer, etc.), and individual- and country-level economic losses (Federal Trade Commission, 2012; World Health Organization, 2016). Apart from these traditional media with their food marketing effects, social media use has exponentially increased among people of all age groups, particularly adolescents (eMarketer, 2016). As the popularity of social media soared, marketers substantially shifted their attention and budgets from television and other traditional media to social media (Hawkes, 2014) including influencer marketing. In fact the main target group of social media influencer marketing is young consumers including adolescents (Aguiar and van Reijmersdal, 2018). A report by the Federal Trade Commission in 2012 previously revealed that US food marketing spending on digital media has doubled, while spending on television advertising dropped by 19.5% (Federal Trade Commission, 2012). As such, social media food marketing has achieved increased sales and preference of advertised products (World Health Organization, 2016).

Adolescents are at a unique life stage that makes them highly sensitive to social influences (Rudan, 2000; van Dam and van Reijmersdal, 2019). They are becoming more independent decision makers, yet at the same time their decision-making processes are limited by inadequate inhibitory control and heightened impulsivity (Pechmann et al., 2005). Moreover, the advertising literacy of this age group, including knowledge of marketing strategies, identification of persuasion intent, and skepticism toward marketing, remains low as compared to that of adults (Boush et al., 1994; Rudan, 2000; Wright et al., 2005; van Dam and van Reijmersdal, 2019). This makes adolescents especially vulnerable to marketing strategies on digital media (e.g., Folkvord et al., 2014). In fact, social media (and its different players of peers, influencers, marketers, etc.) play an important role in shaping the behaviors of adolescent users. According to Story et al. (2002) theoretical framework, adolescent eating behavior is a result of individual (e.g., food preferences, and lifestyle), social environmental (such as peers), physical environmental (such as community settings) and macrosystem influences (e.g., media and social norms). Social media present an unfiltered and hardly monitored ground for the aforementioned different levels of influences. Social media includes peers (whose influence is considered one of the most significant determinants of adolescent behavior), influencers (who are of similar age and perceived as approachable peers), advertisements, media, and norms, all considered as recognized determinants of adolescent behavior (Rudan, 2000; Beaudoin, 2014). Adolescents today are heavy users of multiple social media platforms (Rudan, 2000; Marez, 2018). One of the most popular platforms among adolescents, i.e., Instagram, is also the most frequently employed platform for influencer marketing (Influencer Marketing Hub, 2019).

Despite the presumed influence of such social media messages, particularly on adolescents, little is known about the social media (food) marketing adolescents are exposed to. In response to this imbalance, the World Health Organization called for research on food marketing targeting adolescents, particularly digital media marketing (World Health Organization, 2016). This study therefore aims to explore the food messages adolescent (12–18 years old) consumers encounter when using social media.

A review of the literature shows that there seems to be a strong presence of non-core and branded food products on social media. It is estimated that adolescents see over 9000 food marketing posts on social media every year (Potvin Kent et al., 2019). Holmberg et al. (2016) analyzed the content of images shared by 14-year-old adolescents on Instagram and found the majority were food images, especially of non-core food. Holmberg et al. (2016) found adolescents commonly associated these social media images of branded food with social contexts (e.g., eating with friends at a certain food chain) attaching social meanings to branded products and constructing how adolescents interact with food. In this study, we aim to assess the social media presence of both core and non-core food as well as the sources of these images, including peers, food companies, and influencers. Accordingly, the first research question (RQ1) this study will address is: **What social medium is the most prominent source of food messages that adolescents encounter?** The second research question (RQ2) is: **To what extent do the food messages encountered by adolescents on social media depict core and non-core food?** To answer these questions, a quantitative content analysis of the food messages adolescents are exposed to and their sources will determine the type of foods promoted to adolescents, the social media platforms this food is promoted on, and the sources of these posts.

Recent social media advancements have provided food marketers with platforms to reach out to potential consumers in ways that are perceived more personal and authentic compared to classical media advertising (Lou and Yuan, 2019). Such personalized and borderless social media platforms allow marketers to easily use *owned media* (traditional marketing where a food company posts on its own social media account to its followers), *paid* media (traditional marketing where a food company buys exposure such as a promoted Instagram post), and *earned media* (word-of–mouth) marketing strategies (Stephen and Galak, 2012). Most notably indeed, paid and non-paid influencer and peer posts on social media are a recent game changer in the marketing to younger target audiences. Social media influencers are often perceived as part of the consumers’ network, and their impact is expected to be bigger than a typical celebrity food endorser appearing in a traditional paid advertisement (Lou and Yuan, 2019). As such, the third research question (RQ3) in this study is: **How prevalent are owned, paid, and earned media marketing on adolescents’ social media feeds?** A content analysis will be carried out to explore food marketing strategies used to influence adolescents on social media.

Who are social media influencers? There is no one standard definition to describe a social media influencer, but all definitions share the concept that an influencer is “*an individual or a group of individuals who built their own audience through social media platforms*” (Gross and Wangenheim, 2018, p. 2). As such an influencer is anyone who influences the attitudes and behaviors of other individuals through social media (Freberg et al., 2011; Gross and Wangenheim, 2018). Social media influencers are “cyber-celebrities,” who gain their fame on social media through the textual and visual narration of their personal lives (De Veirman et al., 2017). Similar to traditional celebrities, influencers use their increased social influence to promote branded products (McCracken, 1989; Chatterjee, 2011). Their promotional posts may be subtle, such as broadcasting branded content on their social media feeds, or explicit, such as inviting their followers to repost their branded information (Abidin, 2016). These social media posts likely have an influence on food preferences and behaviors. Unfortunately, when an influencer is portrayed with an unhealthy snack, this leads to higher intakes of non-core foods and higher overall energy intakes compared to when no food is portrayed (Coates et al., 2019b). In a study with pre-adolescents, exposure to messages of social media influencers with branded unhealthy snacks led pre-adolescents to favor the branded snack to a non-branded alternative (Coates et al., 2019c). Food marketing by social media influencers thus seems to have some influence on food preferences and behaviors.

Influencer marketing has grown over the years, and has become a hype among marketers today. Influencer marketing was a 4.6 billion dollar industry in 2018, a figure that is expected to further increase in the future (Influencer Marketing Hub, 2019). Marketing scholars perceive social media influencers as a new, more efficient and influential marketing tool compared to traditional advertisements (De Veirman et al., 2017). The return on investment of influencer marketing is believed to be eight times higher than more traditional types of paid advertising (Influencer Marketing Hub, 2019). Although there might be similarities between traditional advertising and influencer marketing, the latter can be considered a disruptive evolution in marketing for multiple reasons. Social media influencers connect with their followers and offer a new marketing approach that is perceived as engaging and authentic (Lou and Yuan, 2019). Followers believe influencer endorsements represent a public display of the influencer’s intrinsic taste, rather than a violation of their followers’ trust (McQuarrie et al., 2013). Furthermore, viewers and followers identify with influencers as the relatable girls or guys next door, and as such perceive influencers as a credible source of information (Balaban and Mustatea, 2019). For example, in contrast to inaccessible traditional celebrities, influencers engage in direct conversations with and connect with their followers through their social media (Balaban and Mustatea, 2019). Moreover, influencers often establish themselves as experts in specific domains, adding to their credibility as sources for product promotion in the eyes of their followers (Balaban and Mustatea, 2019). For instance, a fitness model might be perceived as a credible source of information on the topic of protein shakes. Finally, influencers generate high returns-on-investments in comparison with celebrities, who typically ask exorbitant fees (Lou and Yuan, 2019).

With the rise of influencer marketing, challenges have surfaced including questions about marketing disclosure of promoted products on social medial. In some developed countries, clear guidelines exist concerning disclosure of influencer marketing. These guidelines are established by governmental organizations, like the Federal Trade Commission in the United States and the Competition and Markets Authority in the United Kingdom (Federal Trade Commission, 2013; Competition and Markets Authority, 2019). These guidelines generally require influencers to clearly disclose the marketing nature of branded content whenever they receive money or any other type of compensation from the food brand (Federal Trade Commission, 2013; Competition and Markets Authority, 2019; Stichting Reclame Code, 2019). For example, the American guidelines state that influencers must disclose any relationship (business, familial, free gifts, etc.) with an endorsed brand clearly in the first three lines (before the “more” button) of an Instagram post (Federal Trade Commission, 2017). Despite the existence of such guidelines, and despite sanctions for non-compliance, approximately 89% of influencers’ social media posts are insufficiently transparent (Influencer Marketing Hub, 2019). Accordingly, the fourth research question (RQ4) this study will explore is: **To what extent do social media influencers, encountered by adolescents on social media, disclose the marketing nature of branded food contents?**

Scholars and marketers alike have faced difficulties in classifying social media influencers, and there has yet to exist standard influencer classification method in the literature. Different sources classify influencers based on motivations, reach, likability, perceived credibility, perceived influence, etc. For example, Gross and Wangenheim (2018) differentiate between influencers, based on their motivations, into four types: Snookers who have no fame or marketing purposes but are there to create and share content and experiences with others, informers who aim to share knowledge and expertise, entertainers whose main aim is to provide their audience with amusement, and infotaineers who aim to draw their audience in with both expertise and amusement. De Veirman et al. (2017) highlighted the role of influencer likability in the classification of influencers, and found that the number of followers can also be used as an indication of influencer likability. However, higher number of followers is not always an indication of an influencer’s success in changing attitudes and consumption behaviors. The identification, success and impact of an influencer may also be measured by the quality of bond an influencer has with their followers (Freberg et al., 2011; Aguiar and van Reijmersdal, 2018). As such, influencers with a smaller number of followers (e.g., less than 1000 followers) probably establish stronger bonds with their audiences as their followers are most likely composed of friends and family. Hence, connection, authenticity and credibility is highly established in such intimate setting, which translates into higher persuasiveness and influence power. Thus, the lines between a highly persuasive and non-highly persuasive influencer are somewhat blurred.

The difficulty of classifying influencers has driven many to also classify influencers based on their number of followers (De Veirman et al., 2017). However, this classification method has yet to be standardized in the literature (including gray literature) (Williams et al., 2017; Linqia, 2019). Aguiar and van Reijmersdal (2018) adapted Williams et al.’s (2017) classification of six types of influencers including: Everyday influencers who have 1–1,300 followers that mainly constitute of family and friends, brand advocates who are satisfied consumers with one or more followers and share their positive experiences with audiences, micro-influencers have 1,000–20,000 followers and typically monetize their accounts, professional (meso) influencers who have 20,000–100,000 followers, macro-influencers are represented by talent agencies and have 100,000–300,000 followers, and celebrity influencers have more than 300,000 followers.

Peer-to-peer social media influence might be a major factor in setting norm beliefs. Research has found that adolescents generally overestimate their peers’ unhealthy food intake, as well as underestimate their peers’ healthy food intake, and that these norm beliefs subsequently predict their own food intake (Lally et al., 2011). Earned social media posts could reinforce, or could even be partially responsible for these misconceptions that adolescents hold about their peers. For instance, seeing peers eat unhealthy or large amounts of food could reinforce the conception that this is normal or appropriate eating behavior. These misconceptions could subsequently lead adolescents to engage in unhealthier eating behaviors themselves. Moreover, influencer marketing could also influence these norm perceptions. Influencers are often seen as relatable peers who share (certain) common interests and attitudes with their followers. Following the similarity/attraction theory (Byrne, 1997), which states that people are attracted to and influenced by people with whom they share common interests, influencers could be an important source of social influence. Finally, this study will attempt to answer the research question (RQ5): **What are the food norms communicated on social media via different types of social media influencers?** A qualitative and quantitative content analysis will determine the presence of different influencers on social media and the food norms (specifically pertaining to food quality and quantity) they typically communicate with their adolescent followers.

## Materials and Methods

### Participants

To attain an in-depth understanding of the food messages that adolescents are continuously exposed to, we carried out a diary study with Flemish adolescents who took screenshots of food images they encountered on their social media platforms for the duration of one week. Adolescents, between the ages 12–18 years old, participated. Officially, the ownership of social media accounts is allowed for those 13 years of age and older, nonetheless in reality adolescents younger than 13 years old manage to access social media, by accessing a kin or friend’s account or by providing incorrect birthdate information when opening a social media account (Ofcom, 2017; Coates et al., 2019a). As such, the inclusion of 12 year-old adolescents in this study was deemed appropriate. Twenty-one adolescent participants, including 11 males and 10 females, were sampled conveniently from different cities across Flanders, Belgium. In preparation for the study, a pilot was held with four Flemish adolescents who shared their social media images for 1 week.

### Procedure

The diary study design is commonly applied in both the nutrition and marketing domains (Vereecken et al., 2008; Siemieniako, 2017), and as such was found appropriate for this study. The study protocol was approved by the first author’s university designated research ethics board. For adolescents under 16 years of age, parental consent was obtained digitally via email or in person via a signed consent form. Participant assent/consent and signatures were obtained in person. Participants also completed brief surveys before, during and after the diary study. The surveys assessed participants’ general social media use, as well as additional aspects of the shared food images such as image source and marketing strategy. The diary study included a one week period of data gathering, considering that this captures the variation in social media use between weekdays and weekends.

For the diary study, participants were trained to take screenshots of any food related images (including images of food, brand logos, restaurants, etc.) they receive or/and engage with on social media. Adolescent participants used software applications, designed for the purpose of this survey, to upload food-related images encountered on the different social media platforms they happened to be using during that one week period. Participants were also asked to take screenshots of images as soon as they are encountered and upload these images at a time and place of convenience (e.g., areas with WiFi access), to reduce potential compliance barriers or study effect on social media usage. Once an image was uploaded, the participant was prompted to answer questions about the image source. Two daily reminders were also programed on participants’ phones to increase participant compliance. Following the completion of the diary study and exit interview, participants were compensated for their weeklong efforts with a monetary voucher in the amount of 25 euros.

### Materials

Applications were designed for the purpose of this research and to facilitate the image sharing process from participants’ smartphones. To accommodate for different phone softwares used by adolescents, two tools were developed. For android users, an application was developed using MIT App Inventor (Massachusetts Institute of Technology, 2018). This application was downloaded and installed on the participant’s phone, and a participant ID number was created and programed into the application. For Apple users, a survey link was developed on Limesurvey (Limesurvey GmbH, 2003).

A short survey was built into the phone tool with the purpose of gaining a better understanding of the context of the shared photos. The first question inquired about the social media platform on which the image was found, a list of response choices was provided; Instagram, Facebook, YouTube, Snapchat, Twitter, or other. The second question assessed the source of the image, with the following response options: “A link or post by a friend, a link or post by a celebrity, a webpage owned by a food brand/company/chain, an ad by a food brand/company/chain (on webpages other than their own), or other.” Finally, participants were asked whether they actively searched for the shared image or they unintentionally came across it.

The entry survey was administered before the one week diary study, and included demographic questions as well as social media use (open-ended) questions. The exit survey was administered following the completion of the one week diary study, with the main purpose of assessing participant compliance during the diary study period. The survey questions inquired about the extent of attention allocated to identifying food related photos during the study period, the extent to which participants believed they uploaded all food-related images they encountered, etc.

### Analysis

We followed thematic content analysis to describe the themes, and interpret underlying messages found in the gathered images (Braun and Clarke, 2006), as has been applied in other similar studies (Roberts and Pettigrew, 2007). Following a review of the literature and an initial revision of the gathered images, the coding guide was developed, and then piloted and further revised with a sample of 32 gathered food images (a subsample of the total images collected during the study). Using the guide, we coded each image to determine any food that is completely or partially presented in the image, and its brand when branded. We further classified the food as core or non-core following the definitions of Kellet et al. (2014) and Toumpakari et al. (2016) who classified core food as that belonging to the dietary guidelines main food groups: fruits, vegetables, cereals, meat and alternatives, as well as milk and alternatives. Food not belonging to any of these groups and that is high in energy yet low in nutrients such as candy, fried chicken, soft drinks, and cakes was classified as non-core food (Kellet et al., 2014; Toumpakari et al., 2016). Additionally, we classified the portion size of food portrayed in the image based on international nutritional guidelines (European Commission, 2019) as either regular when corresponding to the serving sizes recommended per person per meal or excessive if the portion presented exceeds that amount.

Images were also coded for the presence of influencers. To identify and classify social media influencers, we adapted the classifications of Williams et al. (2017) and Aguiar and van Reijmersdal (2018), who classified influencers based on number of followers, monetary compensation, and other key characteristics (such as family, peers, and reach). We additionally modified these classifications to account for whether an influencer’s popularity was found on social media or traditional media (Nouri, 2018). As such we classified social media influencers into 3 main groups: (1) everyday influencers (1 – 1300 followers made up mostly of family and friends), (2) micro-, professional, macro and celebrity influencers who found popularity on social media, (3) celebrity influencers who found popularity on traditional media. To acquire the number of followers an influencer has, we searched for the social media account name (apparent in the image) in its correspondent social media platform in Instagram, Facebook, and YouTube. We then accessed publically available information for that account to determine its number of followers. For a detailed description of variables used in the coding guide and analysis, refer to Table 1.

**TABLE 1:** Description of the coding guide.

**Variable**	**Description**
Food depicted	An exact description of the food portrayed in the image.
Core/non-core	Core food is part of the five main food groups under dietary food guidelines.
Quantity	Regular is equivalent to one food serving per person and excessive is any other portion size exceeding that.
Social context	Showing food in association with a social context including hanging around friends (if the picture clearly shows more than one friend or several plates with friends tagged), celebrating events (any reference to celebration in text or image), spending time with family, hanging with a gathering of people (a larger group of people that extends beyond friends and family), shopping (e.g., at supermarket), and eating at restaurant.
Marketing	Images that show obvious food marketing (not as a subjective interpretation but as a fact).
Purpose	Purpose of the picture: promotion, sharing life moments (sharing food is a sub category of sharing life moments), artistic, entertainment.
Brand	Images that show full or partial brand character/name
Influencers	Based participant’s responses to the diary study multiple choice question enquiring about image source, and additional information tracked from the shared image, influencers were divided into: everyday influencers (average social media users with less than 1,300 followers), micro-, professional, macro- and celebrity influencers who gained popularity via social media, and who use their popularity to influence their followers via social media, and celebrity influencer who first gained popularity outside social media but through television and other traditional media.
Number of followers	Number of followers on Instagram, Facebook, or YouTube. For snapchat and other social media platforms, it was not possible to determine number of followers.
Participant gender	Participants were classified as females or males based on the entry interview responses.
Social media platform	The social media platform, where the image was found, was determined based on the entry interview responses and the information available from the shared image.
Image source	The party who posted the image was determined based on the entry interview responses and the information available from the shared image.

Guided by the coding book, two authors coded each of the images. The codes were then checked for agreement and discrepancies were resolved among the research team. To assess inter-rater reliability, Cohen’s kappa statistic was performed in SPSS version 25 (IBM Corp, 2017). Excluding the branded variable which reached a strong agreement (κ = 0.872, *p* = 0.000), there was moderate agreement on all variables prior to discussion (κ = 0.678, *p* = 0.000 for core/non-core, κ = 0.754, *p* = 0.000 for quantity, κ = 0.698, *p* = 0.000 for social context, κ = 0.760, *p* = 0.000 for marketing, and κ = 0.641, *p* = 0.000 for purpose). Following discussion, all variables were at an almost perfect agreement with κ > 0.900, *p* = 0.000. Codes were recorded in a matrix and analyzed for frequencies and recurring patterns and themes, including food norms depicted, specifically nutritional value (core vs. non-core) and quantity of portrayed food, social contexts associated with food, presence of branded food, obvious marketing strategies in the promotion of branded food, marketing disclosure and intentions behind posted food images. Stata 14 software was also used to perform Chi square analysis (StataCorp, 2014).

## Results

Twenty one Flemish adolescents (11 males and 10 females) between the age of 12 and 18 years old from different regions across Belgium participated in the diary study. The participants were mostly native Belgians (a few were migrants), enrolled in secondary school (only one was a first year university student), and living in urban areas (only 2 participants lived in areas intermediate between urban and rural). All participants reported high use of social media. Participants reported owning at least three social media accounts, while some reported owning up to six social media accounts. At the date of the entry survey, participants reported accessing at least two social media platforms in the past 30 days. Platforms most frequently reported were Instagram (*n* = 19), Facebook (*n* = 18), and Snapchat (*n* = 18). Participants also reported accessing YouTube (*n* = 8), WhatsApp (*n* = 8), and Twitter (*n* = 2).

Adolescent participants took screenshots of food images they encountered on their social media platforms for the duration of one week. They shared a total of 638 images, an average of 30 images per participant (minimum of 2 images submitted by a participant and a maximum of 141 by another participant). Following the cleaning and removal of duplicate images (images uploaded more than once and showing the same time stamp), images that did not show any food content (those that showed food that could not be identified were retained), or images not originating from social media, we were left with 612 images. Only five images were originally posted by the participants themselves, the remaining 607 were images that participants received or/and engaged with on social media.

### Social Media Platforms as Sources of Food Messages

To answer the first research question, we assessed the social media platforms participants used and determined which of these platforms are the most prominent sources of food messages. The aforementioned social media access distributions were also reflected in the images gathered from the participants: 69% of the shared images were acquired from Instagram (*n* = 421), 18% were from Facebook or Messenger (*n* = 110), 9% were from Snapchat (*n* = 54), and 3% from YouTube (*n* = 20). Some food images were also found on WhatsApp (*n* = 3), Weheartit (*n* = 2), Pinterest (*n* = 1), and tumbler (*n* = 1). Interestingly, there were significant differences between male and female participants regarding the social media origin of the shared image [*c*^2^(4) = 105.996, *p* < 0.000]. Female participants were significantly more likely to share food images from Instagram and Snapchat as compared to male participants. On the other hand, male participants were significantly more likely to share YouTube and Facebook food images as compared to females.

### Food Messages Communicated on Social Media

The majority of images shared by participants included food (*n* = 589), while 24 images exclusively showed logos of fast food or drink companies (e.g., Coca-Cola or McDonald’s) without showing actual food. One image only depicted the name of a restaurant, without showing any food or logos. The authors were not able to identify foods visible in four images.

#### Food Norms

##### Core vs. non-core food

Generally, food norms on social media favored the consumption of non-core food in larger quantities (see Table 2). About 67% of the images (*n* = 409) exclusively depicted non-core food such as soft drinks, cake, fries, pizza, sweets, etc. Almost one third of non-core food images (*n* = 143) showed non-core food in regular quantities, whereas more than two thirds of the images (*n* = 233) portrayed non-core foods in excessive quantities. Core and non-core food were also combined in some images. About 10% of the images (*n* = 58) depicted a mix of core and non-core foods in excessive quantities of both (*n* = 23), regular quantities of both (*n* = 22), or regular quantities of core food but excessive portions of non-core food (*n* = 11). On two occasions, the images presented larger portions of core food next to regular portions of non-core food.

**TABLE 2:** Percentages of different food categories depicted in regular or excessive portions, in association with a social context, and which are branded.

	**Core food (*n* = 137)**	**Non-core food (*n* = 409)**	**Combination of core and non-core food (*n* = 58)**
**Portion sizes^∗^**			
Regular	70% (*n* = 96)	35% (*n* = 143)	38% (*n* = 22)
Excessive	28% (*n* = 39)	57% (*n* = 233)	62% (*n* = 36)^∗∗^
**Associated with a social context**			
Yes	37% (*n* = 51)	50% (*n* = 205)	65% (*n* = 38)
No	63% (*n* = 86)	50% (*n* = 204)	35% (*n* = 20)
**Branded**			
Yes	22% (*n* = 30)	57% (*n* = 235)	36% (*n* = 21)
*Owned media marketing*	*1% (n* = *2)*	*6% (n* = *24)*	*2% (n* = *1)*
*Paid media marketing*	*6% (n* = *8)*	*26% (n* = *105)*	*24% (n* = *14)*
*Earned media marketing*	*10% (n* = *14)*	*26% (n* = *108)*	*22% (n* = *13)*
No	78% (*n* = 107)	43% (*n* = 174)	64% (*n* = 37)

*^∗^Some food messages were only depicted as a logo or part of a branded message, as such no portion sizes were associated with these images. ^∗∗^Excessive quantities of at least one type of food.*

Of the 612 images, only 137 images (22% of total images) exclusively depicted core food without showing non-core food. Core food messages included images of meat, fish, rice, pasta, bread, potatoes, eggs, fruits, vegetables, etc. The images varied in characteristics, including shared home or restaurant meals, news or entertainment information, recipes, ads, artistic displays of food, etc. The majority of core food images (*n* = 96) showed regular quantities of core food, while about 30% of those images (*n* = 39) showed excessive quantities of core food. However, excessive quantities were mostly of meat, poultry or starches (rice/pasta/bread) and rarely of vegetables.

##### Food is associated with a social context

Almost half of the images (48%, *n* = 296) attached food to a social context. Often these images depicted images of food associated with spending time with friends (*n* = 119) either by clearly presenting friends in the image, tagging friends to the food image, or both. Many of the images (*n* = 100) contextualized food as part of eating at restaurants with or without friends, family, etc. The association of food with celebration was common as 84 images showed food as a way of celebrating holidays and birthdays.

#### Food Marketing Strategies on Social Media

Almost half of the gathered images (47%, *n* = 289) depicted branded food products. Almost half of these images (*n* = 138) appeared as unpaid word-of-mouth (earned media marketing), however it is highly likely that a big portion of theses food messages (*n* = 50) were in fact paid media marketing. Looking at the images as such, it was difficult to determine the true type of marketing strategy behind these food messages, particularly images posted by influencers and celebrities. More than 43% of the branded food messages, were clearly presented as paid marketing messages (*n* = 126). A smaller number of food messages (*n* = 27) belonged to owned media marketing. Thus in answer to our third research question, we found that earned and paid media marketing were to a great extent prevalent on adolescent’s social media feed, while owned media marketing in comparison was less prevalent.

### Social Media Influencers

#### Presence of Social Media Influencers

##### Everyday influencers

About 27% of the images (*n* = 164) were posted by everyday influencers (mainly peers). These individual social media account holders had smaller numbers of followers and did not seem to benefit from fame or paid marketing. The majority of these everyday influencer images (*n* = 153) were posted on Instagram.

#### Micro-, Meso-, Macro-, and Celebrity Influencers Who Found Popularity on Social Media

Almost 15% of the food images shared by adolescent participants were images posted by or featured either a micro-, meso-, macro-, or celebrity influencer who found popularity on social media (*n* = 86). There was a significant difference between female and male participants in the number of influencer images they shared [Pearson χ^2^(1) = 4.503, *p* = 0.034]. Seventy one influencer social media images were shared by female participants as compared to 15 images only by male participants. As for the social media platform where the images were posted, the majority of influencer food images were posted on Instagram (*n* = 58), while some images were from Facebook (*n* = 14), YouTube (*n* = 10), or Snapchat (*n* = 3).

Social media images mostly featured celebrity influencers with more than 300,000 (673,998–107,000,000) followers (*n* = 48). Images by macro-influencers (109,000–272,000 followers) were also recurrent, as they accounted for more than 22% of influencer images (*n* = 19). The presence of meso- and micro-influencers was less significant. Only five of the identified influencer images belonged to micro-influencers with 3,627–18,477 followers while 14 images belonged to meso-influencers with 20,400–82,600 followers.

#### Celebrity Influencers Who Found Popularity on Traditional Media

Celebrity influencers who found fame outside of social media were only responsible for about 8% (*n* = 48) of the images in the sample. About 80% of these images were shared by female participants, while 20% were shared by male participants (*n* = 10). Similar to other influencers, majority of celebrity images were posted on Instagram (*n* = 39), while fewer were posted on Snapchat (*n* = 5), YouTube (*n* = 2), or Facebook (*n* = 2).

#### Norms Communicated by Social Media Influencers

##### Everyday influencers

Peers seemed to largely communicate norms promoting the consumption of excessive quantities of non-core foods (see Table 3). They posted an overwhelming amount of non-core food images (17% of total images, *n* = 103) and only a small amount (6% of total images, *n* = 34) of core food or combination of core and non-core foods (4% of total images *n* = 24). These individuals mostly posted images of non-core foods in excessive quantities (*n* = 69), with fewer images showing regular portion size of non-core food (*n* = 30), core food (*n* = 24), or combinations of core and non-core foods (*n* = 10).

**TABLE 3:** Percentages of different food categories by origin of posting (among influencers).

	**Core food (*n* = 137)**	**Non-core food (*n* = 409)**	**Combination of core and non-core food (*n* = 58)**
Everyday Influencer	25% (*n* = 34)	25% (*n* = 103)	41% (*n* = 24)
Micro-, meso-, macro-, and celebrity influencers- popularity found on social media	17% (*n* = 23)	14% (*n* = 56)	9% (*n* = 5)
Celebrity influencers- popularity found on traditional media	12% (*n* = 17)	6% (*n* = 23)	12% (*n* = 7)
Other	46% (*n* = 63)	55% (*n* = 227)	38% (*n* = 25)

Seventy-five percent of the images (*n* = 123) posted by everyday influencers were socially contextualized. The most recurring social context found in these images was enjoying food with friends (*n* = 60). This was typically portrayed in association with non-core foods (*n* = 41), while fewer images showed core food (whether exclusively (*n* = 9) or in combination with non-core food (*n* = 9) enjoyed with friends. Another recurring social context was eating out at a restaurant, cafe or any other food establishment (*n* = 49) with or without friends. Similar to contexts of socializing with friends, eating out at restaurants and cafes was mostly recurrent in association with non-core food (*n* = 28), while fewer images contextualized core foods (*n* = 10) and combinations of core and non-core foods (*n* = 11) as part of enjoying a good time at a restaurant or cafe. The third most common social context theme associated with food was celebration (*n* = 29). Similar to other identified social contexts, celebration was mostly associated with non-core food where 16 images showed people celebrating holidays, birthdays, and other events with high-energy low-nutrient foods such as cakes, cookies, alcohol, etc. Fewer celebration images included a combination of core and non-core foods, and only five images of social celebrations exclusively highlighted core foods. Other less recurrent social contexts associated with food included eating linked to gatherings (*n* = 2, 1 non-core food and 1 combination of core and non-core foods), and eating with one’s family (*n* = 1, a combination of core and non-core foods).

##### Micro-, meso-, macro-, and celebrity influencers who found popularity on social media

Micro-, meso-, macro- and celebrity influencers who found popularity on social media seemed to reinforce and contribute to the unhealthy food norms spreading on social media. The majority of the images posted by these influencers were of non-core foods (*n* = 56), most of which were depicted in excessive quantities (*n* = 34) while only 19 images depicted non-core foods in regular quantities. Non-core food images by influencers were usually associated with a social context including celebration (10), eating out at a restaurant (8), eating with friends (7), and gatherings (2).

The contribution of this group of social media Influencers to healthy food messages was much less pronounced as compared to junk food. Only 27% of the influencer images depicted core foods (*n* = 23), which were almost evenly divided between regular and excessive quantities. There were five influencer images showing a mix of core and non-core foods, of which two images showed regular quantities of both foods, and another two showed excessive quantities of both. Among the core food images posted by influencers, only two images were associated with a social context, one image depicted friends eating at a restaurant and another image depicted an influencer surrounded by vegetables celebrating an Instagram milestone in which a target number of followers was attained.

##### Celebrity influencers who found popularity on traditional media

Celebrity influencers, who found popularity on traditional media such as actresses and singers, seemed to contribute to both healthy and unhealthy food norms on social media. Almost half the images posted by celebrities exclusively depicted non-core foods (*n* = 23), most of which were in excessive quantities (*n* = 15) while only eight images depicted non-core foods in regular quantities. More than 35% of the images (*n* = 17) posted by celebrities in the sample were exclusively of core foods, which were almost always depicted in regular portion sizes (*n* = 15). Fewer images (*n* = 7) depicted a combination of both core and non-core foods. Celebrities rarely socially contextualized core food in their images (*n* = 3), yet often posted non-core food in association with some social context (*n* = 12) including eating out at a restaurant (7), eating with friends (5), celebration (2), gatherings (1), and family (1).

#### Marketing Is Not Always Disclosed in an Influencer’s Post

Almost half of the images shared by micro-, meso-, macro- and celebrity influencers (who found popularity on social media) were of branded food products (*n* = 34), of which the majority (*n* = 29) were of branded non-core foods. To assess marketing disclosure, the presence of clear marketing strategies (including marketing disclosure text such as ad, sponsored, collaboration, etc.) was compared with the image purpose. Interestingly, only 8 of the 34 images showed a clear and transparent marketing strategy, although 20 images seemed to have an underlying marketing purpose. This observation did not seem to differ among the four different influencer sub-classifications. In addition to marketing, the majority of images seemed to be posted with the purpose of sharing life moments (*n* = 35) or sharing food (*n* = 35). Some images seemed to be centered around cooking a dish or sharing a recipe (*n* = 12). Others were of purely artistic nature (*n* = 8) or posted for entertainment purposes (*n* = 8).

Marketing disclosure also was not consistent among celebrity influencers who found popularity on traditional media. All 16 branded food products posted by celebrities seemed to have a marketing purpose, yet only two images could be clearly identified as marketed food messages. Other than marketing, celebrities often posted food images with the intention to share food (*n* = 19) or life moments (*n* = 18).

## Discussion

This study provides in-depth understanding of the social media messages that adolescents encounter on a daily basis and sheds light on the food norms typically communicated on social media by marketers, peers, and influencers. To attain an in-depth understanding of the food marketing messages adolescents are continuously exposed to, we carried out a diary study with twenty one Flemish adolescents who took screenshots of food images they encountered on their social media platforms for the duration of one week. Despite the participation of more male than female adolescents in the study, male adolescents only shared 27% of the images. One possible explanation could be that males are exposed to fewer food images on social media compared to females. The difference between the two genders could also be attributed to lower study compliance by male participants as compared to females (e.g., paying less attention to food images on their social media or less likely to send the images once they have seen them). However, studies thus far have not found any gender difference in terms of diary study compliance, particularly electronic diaries (Thieden et al., 2006; Lev-On and Lowenstein-Barkai, 2019).

We found that food norms on social media center around eating non-core foods in oversized portions. This seems to be equally promoted by peers, marketers and influencers. We looked at different classifications of influencers and found that regardless of monetary compensation, number of followers, and fame; influencers (including everyday peer influencers) consistently promoted the consumption of non-core foods in excessive quantities. This is in accordance with the content analysis findings of Holmberg et al. (2016) who found adolescents posted more non-core foods images than core food images on Instagram. Moreover, adolescents’ branded food posts mainly included large non-core food brands, such as Coca-Cola, Ben & Jerry’s, Starbucks, and McDonald’s (Holmberg et al., 2016). This could be due to adolescents’ desire to fit in and connect with friends on social media (Boyd and Ellison, 2007), which in turn means adhering to the prevalent food norms on these networks. As demonstrated in this study, adolescents are exposed daily to non-core food messages through a variety of sources. For example, the most popular food brand pages on Facebook predominantly promote non-core foods (Freeman et al., 2014). Non-core brands have been found to mostly promote their non-core food menus on social media, neglecting core alternatives (Vassallo et al., 2018). On Instagram, Holmberg et al. (2016) also found a prevailing presence of brands that aim to manipulate the emotions of users into buying their products. In sum it seems that social media, similar to traditional media such as TV (Boyland et al., 2012) or even the food on display in supermarkets (Aerts and Smits, 2019), is biased in the type of foods promoted. Moreover, social media is also biased, just like many food packages (Aerts and Smits, 2017), in the food portion sizes displayed. This constitutes a worrisome reality given that both phenomena, which have been shown to cause unhealthy consequences, are now reproduced on social media and combined with social dynamics elements.

Influencers, peers and marketers often contextualized non-core food as part of celebrations, socialization with friends, gatherings, family, and enjoying meals at restaurants and coffee shops. This is in line with Holmberg et al. (2016) who found that adolescents commonly associated social media images of branded food with social contexts (such as eating with friends at a certain food chain), attaching social meanings to branded products and constructing how adolescents interact with food. In this study, we found that similar to peers, other influencers (including micro-, meso-, macro and celebrity influencers), regardless of earned or paid marketing strategy employed, also attached non-core food and branded food, to the same range of social contexts as those portrayed by peers.

The marketing intentions of messages posted by social media influencers were not always clear. Influencers identified in this study posted images that did not consistently disclose the paid marketing nature of endorsed branded food. When assessing the social media account where the image originated, one can notice that some influencers disclose the marketing purposes of a food message, some influencers inconsistently disclose marketing (even for the same product), and yet other influencers do not disclose marketing at all. Unfortunately, even when influencers disclose the paid nature of a food message, this does not always show in the adolescent’s social media feed. Many of the study sample images originally did not seem to correspond to paid media marketing until after tracking and fully opening the original post of the influencer. This is due to the hidden ways influencers disclose marketing (e.g., at the end of a long image description) or simply because the full message is not displayed in the participant’s storyline. This calls for stricter regulations on food marketing disclosure by social media influencers. In Belgium, the country where data collection took place, there are no official disclosure guidelines. In 2018, the Belgian Federal Public Service (FPS economy) released guidelines, however they were immediately evoked following complaints from within the communication sector that the guidelines were ‘too strict.’ To date, no new or reformed guidelines have been introduced by the Belgian government, although the industry presented a self-regulatory framework. Stricter rules apply when it comes to any form of advertising products and services such as unhealthy foods, alcohol or gambling to children and adolescents, but it is difficult to demonstrate that such ads are targeting youth. As a result, Belgian influencers typically feel little incentivized to clearly disclose the commercial nature of their posts, and do not receive sanctions for paid posts that are insufficiently transparent. This made it difficult to uncover whether a social media post in which an influencer mentioned or depicted a food brand was paid or earned marketing. However, not all the influencers in this study were Belgian as many were international influencers. Furthermore, most governments have clear guidelines on marketing disclosure by influencers (Federal Trade Commission, 2013; Competition and Markets Authority, 2019; Stichting Reclame Code, 2019). Thus, our findings support reports that indicate a high prevalence of marketing disclosure non-compliance by influencers (Influencer Marketing Hub, 2019). One possible reason for this is that marketers are insufficiently aware of the recent guidelines concerning online marketing disclosure (Linqia, 2019). Additionally, marketing disclosure among influencers is considered difficult due to uncertainties surrounding the nature of situation and posts that constitute marketing, manners in which carry out the disclosure, and the parties responsible for disclosure reinforcement (Aguiar and van Reijmersdal, 2018). This calls for stricter regulations on food marketing disclosure by social media influencers and for efforts to increase awareness of marketing disclosure among social media influencers themselves.

This study highlights the role of social media influencers as a powerful tool in directing food norms on social media, which may in turn influence adolescent cognitions, attitudes and behaviors. Influencers constitute a large share of electronic word of mouth and paid marketing on social media (Evans et al., 2018). They have the advantage of coming at a low cost and spread the message faster than traditional advertising (Evans et al., 2018). The majority of influencers in this study were lifestyle influencers, and only two influencers were food influencers. Yet regardless of focus and niche, influencers of different topics seemed to consistently contribute to the same food norms.

This is one of the few studies that assesses the exposure of adolescents to social media food messages. Although the study included a small convenience sample of adolescent participants, nonetheless this sample was enough to carry out an in-depth analysis of more than 600 images adolescents were exposed to during the course of one week of social media consumption. Note that it is possible adolescents encountered more images during the week of their participation; that they may not have noticed all the food images they were exposed to on social media or that they may not have shared images even after noticing them. To account for this challenge, we instructed participants to send us any image that remotely reminded them of food. We believe this instruction effectively helped participants identify any food-related image necessary for this study, as we received different types of food images including images of empty kitchens and restaurant dining areas (which were dropped in the cleaning process). To maximize participant compliance, we scheduled two daily reminders on participants’ phones and compensated all participants with a monetary voucher from a relevant online vendor. Furthermore, participants reported that they sent food images they encountered on their social media during their 1 week of participation to a great or somewhat great extent. Another limitation in this study was that it was not always possible to determine who posted the image and what (if any) text was linked to that image. However, participants responded to questions about the image source during the study, which made it possible to always determine if the image was sent by a peer, a celebrity/influencer or a food company. Finally, it was not always possible to retrieve all influencer images from social media, especially those on snapchat. However, the images we had were enough to determine the type of food messages communicated and marketing strategies employed.

## Conclusion

This study explores what minors encounter in terms of food marketing when using social media. We highlight the spread of food norms on social media that encourage the overconsumption of non-core foods. Branded non-core foods are promoted via earned, paid and owned media marketing strategies by peers, influencers and marketers alike. Thus we call for reinforced food marketing regulations on social media. This is especially important in the case of paid marketing via influencers, who inconsistently disclose the paid marketing nature of the food messages they share. There is a need for stricter regulations to govern how influencers disclose food endorsements, including a consistent disclosure of every food message posted on social media. Disclosure also must be as clear and obvious to adolescents consumers as the food message portrayed (including for images that show on a minor’s timeline). Furthermore, disclosure could be coupled with advertising literacy to ensure that adolescents truly grasp the monetary motivations of paid influencers.

The food marketing strategies spread on social media may hold an important lesson for health professionals and decision makers. The employment of earned and paid marketing strategies play a big role in the spread of branded and non-branded core foods norms on social media. Accordingly, health decision makers and practitioners may want to consider the recruitment of social media influencers to spread core-food norms on social media. Furthermore, incorporating core food in a variety of artistic photos and entertainment and news posts on social media may encourage word-of-mouth media promotion. Health campaigns may also benefit from contextualizing core food and associating it with social contexts such as eating out with friends, celebrations, spending time with family, etc. To better understand the potential role of influencers in health promotion, we call for research that tests the persuasiveness of prominent social media food messages identified in this study and study the effects of social media food marketing on adolescents. Future studies may also assess adolescents of different backgrounds (e.g., nationalities, marginalized populations, etc.) or the engagements and interaction adolescents have with social media food messages. We also call for research that assesses the effectiveness of influencer marketing and social contextualization in the spread of norms promoting overall healthier lifestyles and eating among adolescents.

## Data Availability Statement

The datasets generated for this study are available on request to the corresponding author.

## Ethics Statement

The studies involving human participants were reviewed and approved by the Social and Societal Ethics Committee (Sociaal-Maatschappelijke Ethische Commissie), KU Leuven. Written informed consent to participate in this study was provided by the participants’ legal guardian/next of kin where required, and participants provided written, informed assent/consent.

## Author Contributions

TS, YQ, and CD contributed to the study conception and design. YQ, NM, LH, and TS contributed to the data collection and analysis. YQ wrote the first draft of the manuscript. LH and NM wrote sections of the manuscript. All authors contributed to the manuscript revision, and read and approved the submitted version.

## Conflict of Interest

The authors declare that the research was conducted in the absence of any commercial or financial relationships that could be construed as a potential conflict of interest.
